# Emergency versus delayed hepatectomy following transarterial embolization in spontaneously ruptured hepatocellular carcinoma survivors: a systematic review and meta-analysis

**DOI:** 10.1186/s12957-022-02832-7

**Published:** 2022-11-18

**Authors:** Wei Zhang, Zhangkan Huang, Xu Che

**Affiliations:** 1 Department of Hepatobiliary and Pancreatic Surgery, National Cancer Center/National Clinical Research Center for Cancer/Cancer Hospital & Shenzhen Hospital, Chinese Academy of Medical Sciences and Peking Union Medical College, Shenzhen, 518116 China; 2 Department of Pancreatic and Gastric Surgery, National Cancer Center/National Clinical Research Center for Cancer /Cancer Hospital, Chinese Academy of Medical Sciences and Peking Union Medical College, 17 Panjiayuan Nanli, Chaoyang District, Beijing, 100021 China

**Keywords:** Hepatocellular carcinoma, Hepatectomy, Transarterial embolization, Rupture, Prognosis

## Abstract

**Background:**

Spontaneous rupture is a life-threatening complication of hepatocellular carcinoma (HCC). Recent trends in surgical treatments avoid emergency hepatectomy (EH) and favor emergency transarterial embolization (TAE) followed by delayed hepatectomy (DH). Still, there is debate on which is the better treatment option and whether delaying hepatectomy increases peritoneal metastasis.

**Aim:**

To provide evidence-based references for the optimal management of patients with spontaneously ruptured HCC by comparing the outcomes of EH and DH.

**Methods:**

Literature on postoperative outcomes of EH and DH in patients with spontaneously ruptured HCC published between the date of the database establishment and May 2022, was identified in the PubMed, EMBASE, and Cochrane Library databases. Revman 5.3 software was used for statistical analyses.

**Results:**

Nine publications were identified, including a total of 681 patients. Of those, 304 underwent EH, and 377 underwent TAE followed by DH. The meta-analysis results indicated that the in-hospital mortality rate in the EH patient group was significantly higher than that in the DH patient group (relative risk (RR) = 2.17, 95% confidence interval (CI) 1.03–4.57, *p* =0.04). There was no significant differences in the rates of postoperative complications (RR = 1.21, 95% CI 0.77–1.90, *p* = 0.40), postoperative hospital stay (WMD = − 0.64, 95% CI − 5.61–4.34, *p* = 0.80), recurrence (RR = 1.09, 95% CI 0.94–1.25, *p* = 0.27), peritoneal metastasis (RR = 1.06, 95% CI 0.66–1.71, *p* = 0.80), 1-year survival (RR = 0.91, 95% CI 0.80–1.02, *p* = 0.11), or 3-year survival (RR = 0.81, 95% CI 0.61–1.09, *p* = 0.17) in survivors between the two patient groups.

**Conclusion:**

The postoperative outcomes of the spontaneously ruptured HCC survivors who received EH were similar to those who received emergency TAE followed by DH. However, the in-hospital mortality rate was higher in EH patients. Based on the findings, DH with TAE first strategy might be considered over EH as the first line treatment modality. However, these findings await further validation by future high-quality studies.

**Supplementary Information:**

The online version contains supplementary material available at 10.1186/s12957-022-02832-7.

## Introduction

Hepatocellular carcinoma (HCC) is the sixth most common diagnosed malignancy and third causes of cancer-related death in the world [1]. In recent years, the incidence of spontaneously ruptured HCC (SR-HCC) has increased, reaching as high as 10 to 15% in some regions of Asia [2, 3]. Generally, the outcome of patients with SR-HCC is poor if not treated aggressively. The key to management is adequate hemostasis and fluid resuscitation to rescue the patient. Currently, the main treatment options are hepatectomy and transcatheter arterial embolization (TAE). Recent literature reported TAE followed by delayed hepatectomy (DH) as the dominant option. However, some patients still require emergency hepatectomy (EH), especially if they are hemodynamically stable and have a resectable lesion, as this might promote hemostasis and potentially curative resection in such cases.

Hepatocellular carcinoma is a highly aggressive tumor, and SR often results in intra-abdominal tumor dissemination, leading to peritoneal metastasis. Whether delaying surgery increases the potential for tumor metastasis and recurrence remains to be clarified. With the widespread use of TAE followed by DH, this concern is growing. Therefore, we conducted a meta-analysis to systematically and comprehensively evaluate the effectiveness of these two approaches in the treatment of SR-HCC.

## Materials and methods

This meta-analysis was reported according to the Preferred Reporting Items for Systematic Reviews and Meta-Analyses (PRISMA) guidelines [4]. This meta-analysis is registered on the PROSPERO website (https://www.crd.york.ac.uk/prospero) under the registration number CRD42020211919.

### Search strategy

We conducted a literature search of the EMBASE, PubMed, and Cochrane Library databases to identify relevant available articles from the database’s inception to May 2022. The keywords for retrieval were liver resection, hepatectomy, rupture, and hepatocellular carcinoma. The retrieval strategies for each database are attached in Supplemental file 1. The logical words, AND and OR, were alternately applied to the keywords. The reference lists of the included studies were reviewed for undetected relevant studies. The functions of “Similar articles” and “Cited by” in the databases were applied to expand the literature retrieval. We contacted the original authors to obtain extra information if necessary. Only the latest study with the largest sample size and the highest quality was selected if some studies were from the same author or research center and the samples included were overlapping.

### Inclusion criteria

(1) Objects: SR-HCC patients. (2) Published literature on the comparison of postoperative survival between EH and DH for SR-HCC, including randomized controlled studies, and prospective or retrospective cohort studies. (3) Sample size: unlimited. (4) Follow-up time: unlimited. (5) Literature language: unlimited. (6) Study type: human study.

### Extraction criteria and quality assessment

(1) Republished studies, unpublished studies, and studies without complete information or valid data, and those where the authors were unavailable. (2) Single-arm EH or DH studies. (3) Other treatments, such as transcatheter arterial embolization only and conservative treatment only. (4) Laparoscopic studies, robotic research, reviews, case reports, and animal experiments. A consensus meeting was held to decide study eligibility if the reviewers disagreed on the inclusion or exclusion criteria of a given study.

The retrieved and included studies were retrospective cohort studies. Quality assessment of the cohort studies was based on the Newcastle-Ottawa Scale (NOS), specifically including population selection, comparability, exposure evaluation, or outcome evaluation. The semi-quantitative star system was used for the quality evaluation of the retrospective literature, with a perfect score indicated by 9 stars (Supplemental file 2).

### Statistical analysis

Revman 5.3 was used in this meta-study for statistical analyses. The Mantel-Haenszel method was used to estimate the combined binary effect (relative risk, RR). The Inverse Variance method was used to estimate the combined effect of continuous data (weighted mean difference, WMD). RRs and WMDs with a 95% confidence interval (CI) were calculated to compare the incidence of postoperative outcomes between the EH group and the DH group. Heterogeneity among the included studies was qualitatively evaluated using a *χ2*-based *Q* test and *P* values of less than 0.10 were considered statistically significant. The level of heterogeneity between studies was evaluated using *I*^2^ statistics. An *I*^2^ value of < 30% was considered to indicate low heterogeneity; 30%  ≤ *I*^*2*^  ≤  50% indicated moderate heterogeneity, and *I*^2^ >  50% represented high heterogeneity. Random models were used in this meta-analysis. Sensitivity analysis was performed by removing one study at a time to assess whether the results could have been markedly affected by the study. The results with less heterogeneity between the studies were selected if the results were reversed after sensitivity analysis. Deleted literature is described in the “Results” section. Begg’s test and Egger’s test were conducted using Stata SE 12.0 to quantitatively evaluate the publication bias of the included studies, with the significance level limited to 0.05 (Supplemental file 3).

## Results

### Search results and study selection

**Fig. 1:**
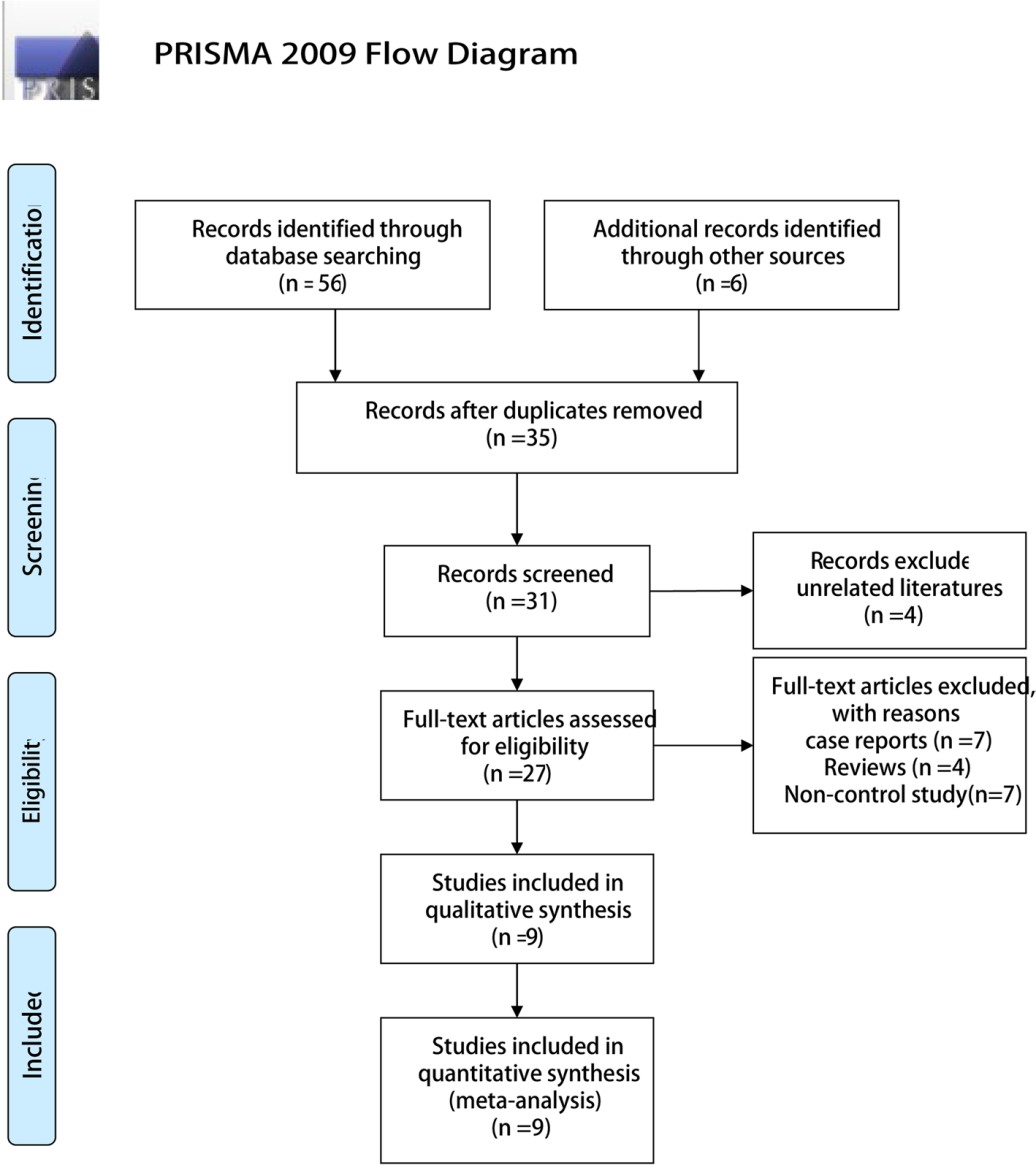
Flow diagram of the literature search process according to PRISMA 2009

A total of 62 articles were retrieved by searching electronic databases and manually searching the relevant reference lists. After duplicates were identified and excluded, 35 articles remained. We then excluded unrelated reviews, case reports, unrelated systematic reviews, and meta-analyses, as well as studies that were clearly irrelevant based on their title or abstract. Nine articles remained. The detailed steps of the literature search are shown in Fig. 1. Nine studies with a total of 681 patients were included in the final analysis. In total, 304 patients (44.6%) received EH, and 377 (55.4%) patients received DH. The characteristics of these studies are presented in Table 1. The clinical characteristics and postoperative clinical outcomes of the two groups included in the study are summarized in Table 2.

**Table 1:** Basic characteristics and quality assessment of the enrolled studies

Study	Country	Type	Period	Case		Age		Sex (m/f)		Quality
				EH	DH	EH	DH	EH	DH	(NOS)
Buczkowski [5]	Canada	R	1985–2004	10	10	51 ± 19	58 ± 13	7/3	8/2	7
Ou 2016 [6]	China	R	2005–2014	73	58	52 (29–77)	57 (31–81)	59/14	45/13	8
Ren 2019 [7]	China	R	2011–2016	17	27	53.6 (26–73)	47.0 (18–70)	14/3	22/5	7
Sun 2013 [8]	China	R	1999–2011	10	9	40.8 ± 11.4	48.2 ± 14.2	2/8	0/9	6
Wu 2019 [9]	China	R	2005–2015	30	100	41.5 ± 11.5	47.8 ± 11.2	25/5	89/11	6
Yang H 2014 [10]	China	R	2003–2012	17	11	48.5 (23–78)	48.5 (23–78)	NA	NA	7
Yang T 2013 [11]	China	R	2000–2009	28	115	49 ± 10	49 ± 11	22/6	105/10	6
Zhong 2016 [12]	China	R	2004–2014	79	27	58.8 ± 6.8	58.3 ± 4.6	24/3	68/11	6
Zhou 2020 [13]	China	R(PSM)	2012–2017	40	20	50% (> 60 years)	42.5% (> 60 years)	18/2	37/3	8

*EH* emergency hepatectomy, *DH* delayed hepatectomy, *R* retrospective study, *PSM* propensity score matching, *NA* not available, *NOS* Newcastle-Ottawa Score

**Table 2:** Clinical characteristics and postoperative clinical outcomes of the two groups included in the study

Study	Tumor sizea^a^		Liver cirrhosis^b^		BCLC stage (A/B/C)		Child–Pugh (A/B/C)		Hemorrhagic shock^b^		UICC stage (I/II/III/IV)		Tumor number (solitary^b^)		Type of surger (Minor/Major)		MVI^b^		Resection margin^b^	
	EH	DH	EH	DH	EH	DH	EH	DH	EH	DH	EH	DH	EH	DH	EH	DH	EH	DH	EH	DH
Buczkowski 2006 [5]	7 ± 4	7 ± 3	7 (70%)	5 (50%)	NA	NA	NA	NA	NA	NA	NA	NA	NA	NA	5/5	4/6	NA	NA	NA	NA
Ou 2016 [6]	10 (4–23)	11 (6–25)	48 (65.8%)	37 (63.8%)	NA	NA	NA	NA	37 (50.7%)	25 (43.1%)	NA	NA	1 (100%)	1 (100%)	56/17	35/23	38 (52.0%)	29 (50.0%)	51 (69.9%)	42 (72.4%)
Ren 2019 [7]	NA	NA	8 (47.1%)	14 (51.9%)	3/9/5	7/12/9	B/C:14/3	B/C:25/2	NA	NA	NA	NA	NA	NA	NA	NA	NA	NA	NA	NA
Sun 2013 [8]	7.1 ± 3.5	7.1 ± 3.3	9 (100.0%)	9 (90.0%)	NA	NA	NA	NA	NA	NA	NA	NA	9 (100.0%)	10 (100.0%)	NA	NA	NA	NA	NA	NA
Wu 2019 [9]	6.5 (4.8–8.5)	8.0 (5.3–10.0)	15 (50.0%)	75 (75.0%)	16/6/7/1	59/20/21/0	B/C:17/13	B/C:90/10	15 (50.0%)	8 (8.0%)	16/6/7/1	59/20/21/0	23 (76.7%)	77 (77.0%)	26/4	87/13	6 (20.0%)	18 (18.0%)	29 (96.7)	97 (97%)
Yang H 2014 [10]	NA	NA	NA	NA	NA	NA	10/7/0	8/3/0	NA	NA	0/15/2/0	0/7/4/0	NA	NA	NA	NA	NA	NA	NA	NA
Yang T 2013 [1]	NA	NA	NA	NA	NA	NA	24/4/0	108/7/0	18 (64%)	14 (12·2%)	NA	NA	NA	NA	NA	NA	NA	NA	22 (79%)	94 (81.7%)
Zhong 2016 [12]	8.8 ± 2.3	9.0 ± 1.4	66 (83.5 %)	20 (74.1%)	NA	NA	53/17/9	15/9/3	NA	NA	15/24/40	1/17/9	45 (57.0 %)	10 (37.0 %)	NA	NA	NA	NA	NA	NA
Zhou 2020 [13]	7.1 ± 3.9	7.3 ± 2.3	31 (77.5%)	15 (75%)	31/6/1	17/3/0	31/9/0	11/9/0	15 (37.5%)	10 (50%)	NA	NA	33 (82.5)	17 (85%)	35/5	17/3	14 (35%)	7 (35%)	35 (87.5%)	19 (95%)

*EH* emergency hepatectomy, *DH* delayed hepatectomy, *NA* not available, *BCLC* Barcelona Clinic Liver Cancer, *MVI* microvascular invasion of liver cancer

^a^ cm, with mean and standard deviation

^b^*n*, %

### Meta-analysis results

Seven postoperative outcomes of patients who underwent EH and DH for the treatment of SR-HCC were analyzed in this meta-analysis, as shown in Table 3. Overall survival (OS) was defined as the interval between the date of surgery and the date of the patient’s death or the end of follow-up. Common complications of hepatectomy, defined by the Clavien-Dindo complication grading system, were extracted for analysis. Recurrence and peritoneal metastasis were judged on the basis of tumor markers such as alpha-fetoprotein (AFP), and imaging data such as computed tomography (CT) and magnetic resonance imaging (MRI).

**Table 3:** Meta-analysis results of all available studies with measured outcomes

Measured outcomes	No. studies	No. patients	Heterogeneity test		Model	RR/WMD	95%*CI*	*P*
			*I*^*2*^*(%)*	*P*				
Hospital mortality	8	286 vs. 351	0	0.64	Random	2.17	1.03,4.57	**0.04**
Postoperative complication	6	154 vs. 132	0	0.8	Random	1.21	0.77,1.90	0.40
Postoperative hospital stays	4	140 vs. 115	87	< 0.0001	Random	− 0.64	− 5.61,4.34	0.80
Peritoneal metastasis	3	122 vs. 101	0	0.9	Random	1.06	0.66,1.71	0.80
Recurrence rate	4	128 vs. 110	0	0.98	Random	1.09	0.94,1.25	0.27
1-year OS	6	154 vs. 132	0	0.8	Random	0.91	0.80,1.02	0.11
3-year OS	4	131 vs. 111	6	0.36	Random	0.81	0.61,1.09	0.17

*No.* number of, *RR* risk ratio, *WMD* weighted mean difference, *CI* confidence interval, *OS* overall survival, statistical significant results are shown in bold

#### In-hospital mortality

**Fig. 2:**
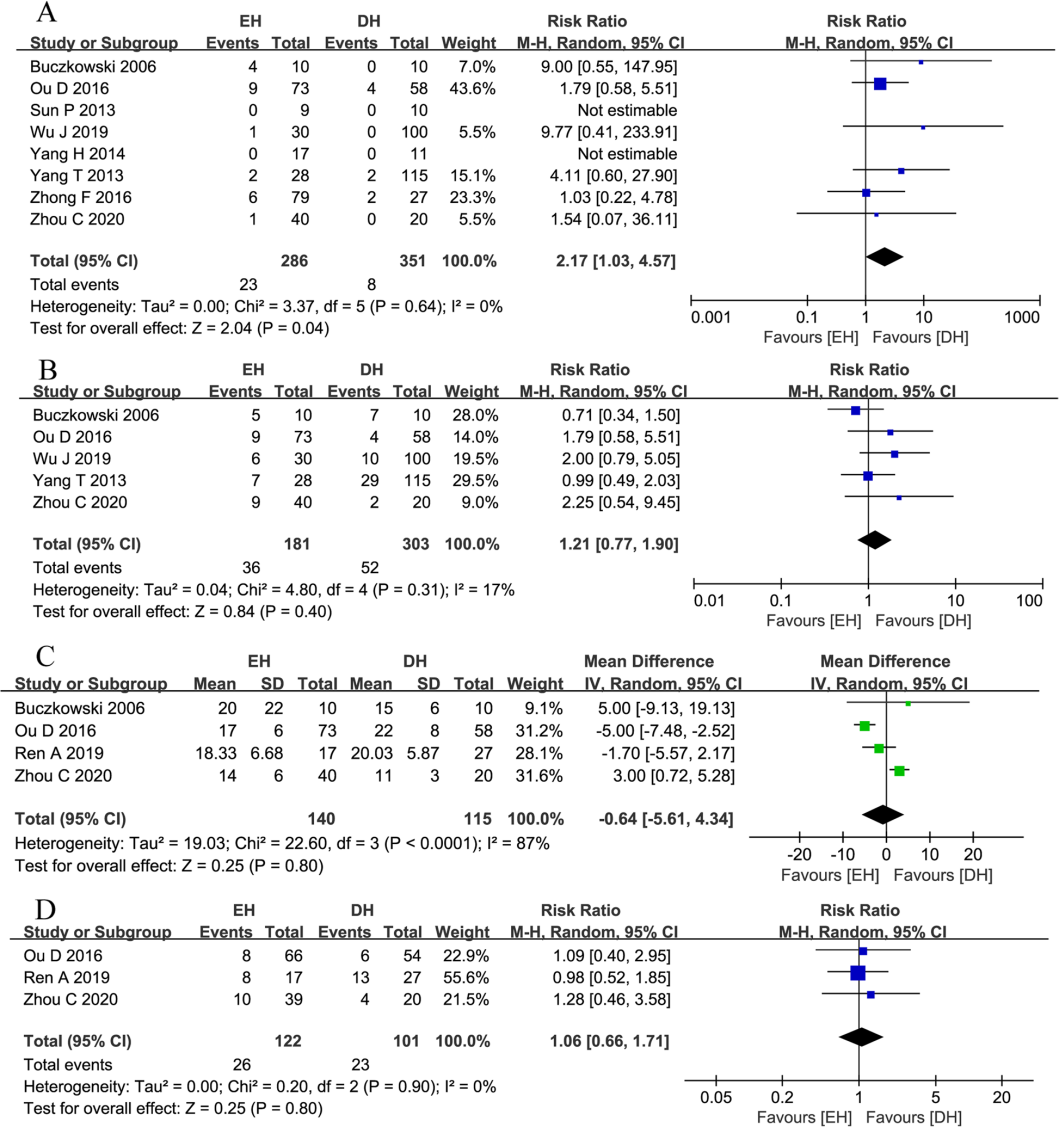
Forest plots of **A** in-hospital mortality, **B** postoperative complications, **C** postoperative hospital stay, and **D** peritoneal metastasis

In-hospital mortality was reported in 8 studies [5, 6, 8–13]. Low heterogeneity was observed among these studies (*I*^*2*^*=* 0%, *p* = 0.64). The random-effect model was applied, and the combined effect was RR = 2.17, 95% CI 1.03–4.57, *p* = 0.04. The in-hospital mortality in the EH group was significantly higher than that in the DH group (Fig. 2A).

#### Postoperative complications

Postoperative complications were reported in 5 studies [5, 6, 9, 11, 13]. Low heterogeneity was observed among these studies (*I*^*2*^ = 17%, *p* = 0.31). The random-effect model was applied, and the combined effect was RR = 1.21, 95% CI 0.77–1.90), *p* = 0.40. There was no significant difference in postoperative complications between the EH group and the DH group (Fig. 2B).

#### Postoperative hospital stay

The postoperative hospital length of stay was reported in four studies [5–7, 13]. High heterogeneity was observed among these studies (*I*^*2*^ = 87%, *p* < 0.0001). The random-effect model was applied, and the combined effect was WMD = − 0.64, 95% CI − 5.61–4.34, *p* = 0.80. There was no significant difference in the postoperative hospital length of stay between the EH group and the DH group (Fig. 2C).

#### Peritoneal metastasis

Peritoneal metastasis was reported in three studies [6, 7, 13]. Low heterogeneity was observed among these studies (*I*^*2*^ = 0%, *p* = 0.90). The random-effect model was applied, and the combined effect was *RR* = 1.06, 95% CI 0.66–1.71, *p* = 0.80. There was no significant difference in abdominal metastasis between the EH group and the DH group (Fig. 2D).

#### Recurrence rate

**Fig. 3:**
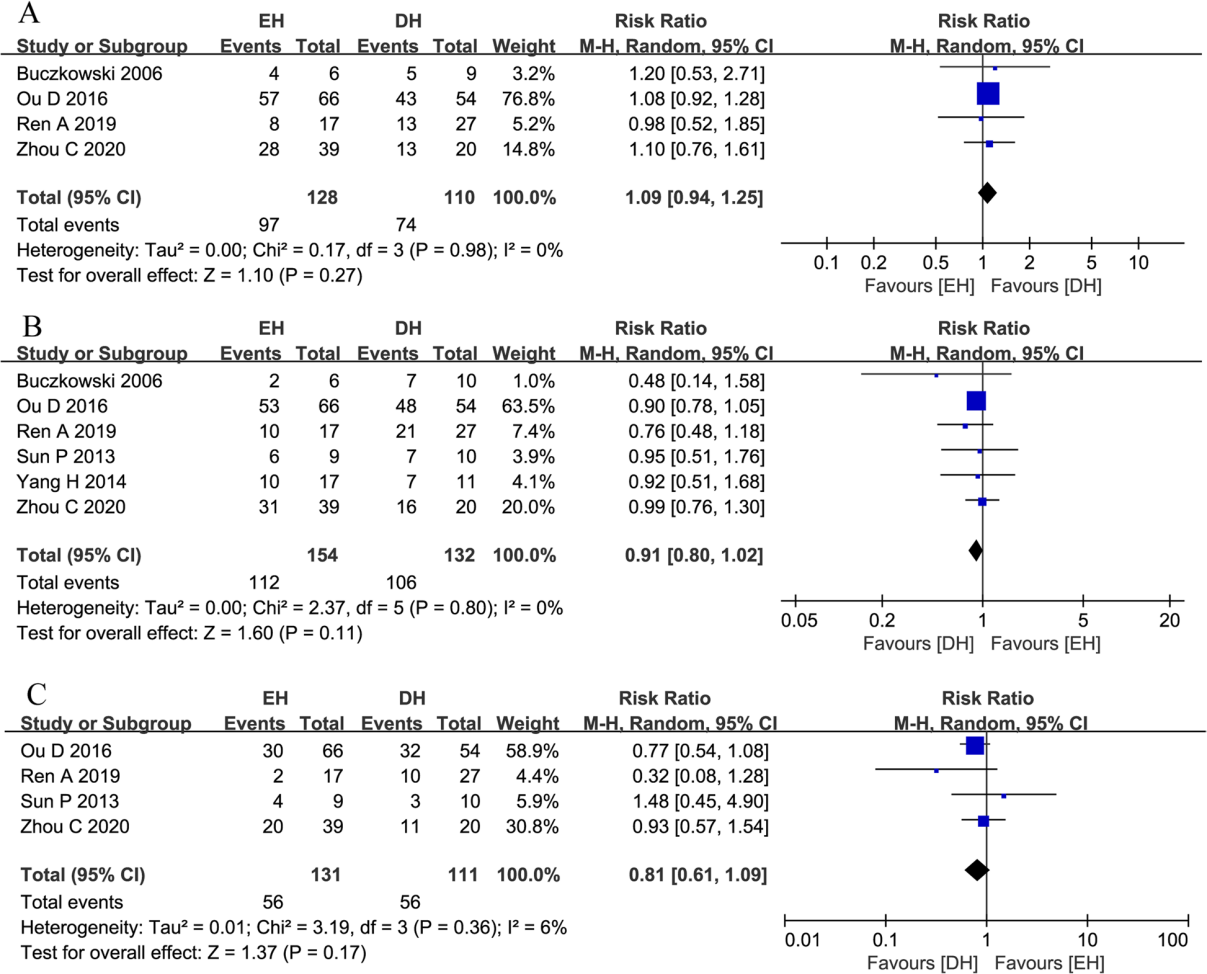
Forest plots of **A** recurrence rate, **B** 1-year overall survival, and **C** 3-year overall survival

The recurrence rate was reported in four studies [5–7, 13]. Low heterogeneity was observed among these studies (*I*^*2*^ = 0%, *p* = 0.98). The random-effect model was applied, and the combined effect was RR = 1.09, 95% CI 0.94–1.25, *p* = 0.27. There was no significant difference in the recurrence rate between the EH group and the DH group (Fig. 3A).

#### One-year OS

One-year OS was reported in six studies [5–8, 10, 13]. Low heterogeneity was observed among these studies (*I*^*2*^ = 0%, *p* = 0.80). The random-effect model was applied, and the combined effect was RR = 0.91, 95% CI 0.80–1.02), *p* = 0.11. There was no significant difference in 1-year OS between the EH group and the DH group (Fig. 3B).

#### Three-year OS

Three-year OS was reported in four studies [6–8, 13]. Low heterogeneity was observed among these studies (*I*^*2*^ = 6%, *p* = 0.36). The random-effect model was applied, and the combined effect was RR = 0.81, 95% CI 0.61–1.09, *p* = 0.17. There was no significant difference in 3-year OS between the EH group and the DH group (Fig. 3C).

### Sensitivity analysis and publication bias

**Fig. 4:**
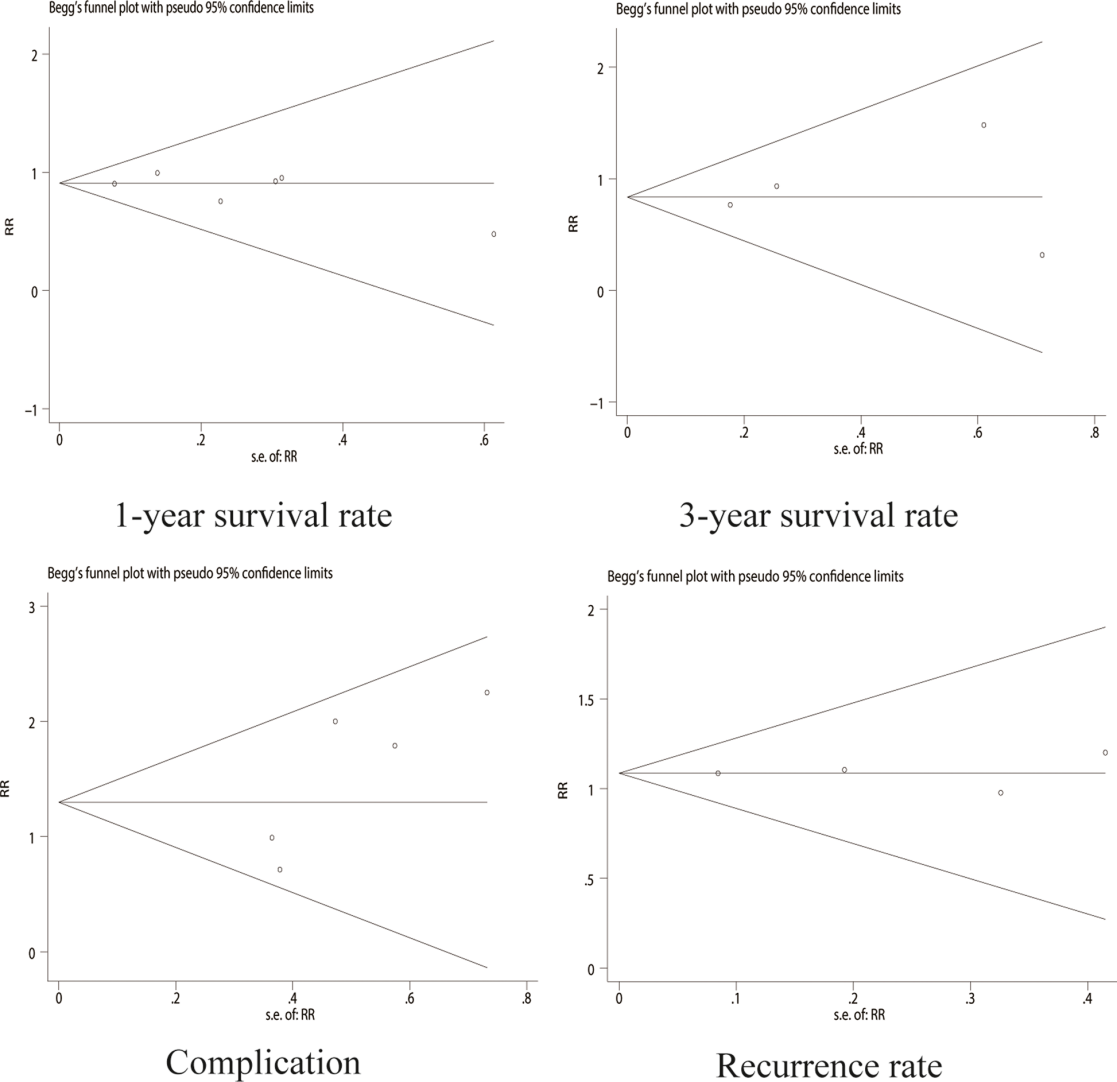
Begg’s funnel plot with pseudo 95% confidence limits

The outcome index results are stable in each meta-analysis when sensitivity analysis is performed. We did not detect publication bias by Begg’s test or Egger’s test (Supplemental file 3). Begg’s funnel plot with pseudo 95% confidence limits is shown in Fig. 4.

## Discussion

Spontaneous rupture of HCC occurs recurrently due to excessive internal pressure or the fragility of the tumor wall. At the time of rupture, HCC might remain asymptomatic, or it can cause bleeding and abdominal pain [11]. Traditionally, the diagnosis has been based on clinical manifestations, such as the sudden onset of pain with shock and intra-abdominal hemorrhage, which could be detected by paracentesis. The diagnosis is often late, and most patients die within 1 month after SR-HCC occurrence [2, 14, 15]. The development of ultrasonography, CT, and angiography has enabled the rapid diagnosis of SR-HCC, which has earned patients valuable treatment time. Hepatectomy is performed in hemodynamically stable patients that exhibit easily resectable lesions, whereas intraoperative tamponade, suture, or separate ligation of the hepatic artery is often used to control bleeding for hemodynamically unstable patients and patients exhibiting unresectable tumors.

Current treatment strategies for SR-HCC avoid emergency surgery and favor TAE followed by DH. In 1993, Dr. Cherqui [16] combined his own experience with the analysis of 250 cases of ruptured HCC in the literature and showed that for patients with resectable and preserved liver function, EH was the preferred treatment method, and if possible, transcatheter hepatic artery embolization should be the next option in high-risk patients or before hepatectomy. Since the year 2000, with the development of radiological interventions, most hospital facilities have been equipped with interventional departments that can complete emergency TAE. TAE followed by DH has been increasingly used for hemostasis in SR-HCC patients [17, 18]. However, SR-HCC increases the risk of extrahepatic recurrence, metastasis, and peritoneal organ involvement, as well as lung, bone, and distant lymph node metastases [19, 20]. Presently, there is no effective treatment, especially for peritoneal metastases. Therefore, concerns have arisen that DH might exacerbate this trend. By analyzing the existing studies, this meta-analysis found no statistical differences in recurrence and abdominal metastasis rates between survivors in the EH group and the DH group.

In this study, we observed that SR-HCC required urgent treatment with patients presenting a trend toward higher in-hospital mortality rates, which could be because this group of patients includes those that might present with extremely advanced and/or aggressive comorbidities. In contrast, patients eligible for DH are selected through earlier management. If the death rate in the EH group is included in the calculation of long-term survival, then a large bias occurs. Therefore, we excluded in-hospital deaths and then evaluated the survival of patients who underwent emergency and DH. Our meta-analysis found no statistically significant differences in 1-year and 3-year survival between the emergency and DH groups. Furthermore, there were no significant differences in postoperative complications or length of postoperative hospital stay due to similar liver resection procedures. Based on the results of this meta-analysis, TAE followed by DH might be considered over EH as the first line treatment modality for SR-HCC.

When undergoing EH, the uncertainty of liver function, hemodynamic instability, and systemic coagulation dysfunction might result in a vicious cycle that worsens liver function and could lead to death. As previously stated, the in-hospital mortality rate in the EH group was significantly higher than that in the DH group. The high-risk factors associated with death should be determined before EH in future studies.

## Conclusion

The postoperative outcomes of SR-HCC survivors who underwent EH were similar to those who underwent emergency TAE followed by DH. However, the in-hospital mortality rate was higher in patients who received EH. Based on current evidence, DH with TAE first strategy might be considered over EH as the first line treatment modality. These findings still need to be validated by additional high-quality and longer follow-up studies.

## Limitations

We acknowledge several limitations to this study. (1) Due to the lack of randomized controlled trials and the fact that all of the included literature was composed of retrospective cohort studies, bias could not be avoided in this meta-analysis. (2) SR-HCC is a relatively rare event. Thus, comparative studies reporting EH and DH are lacking. The number of patients included in the study was small, which may lead to type I or II errors. (3) Although we considered that the emergency management of the DH group was TAE, not all patients in the included studies underwent TAE. Instead, a small fraction underwent emergency surgical hemostasis or conservative treatment. Although this proportion was very low, it is possible that it might have introduced some bias. (4) Except for one study from Canada, the other studies were from China, limiting the applicability of the conclusions of this meta-analysis.

## Supplementary Information

**Additional file 1: Supplemental file 1.** The search formula for each database.

**Additional file 2: Supplemental file 2.** The Risk of bias in the included retrospective cohort studies (by the Newcastle–Ottawa quality assessment tool).

**Additional file 3: Supplemental file 3.** Begg’s test and Egger's test for each outcome.

## Data Availability

All data are generated from public data, which has been shown in the article.

## References

[1] Sung H, Ferlay J, Siegel RL, Laversanne M, Soerjomataram I, Jemal A, Bray F (2021). Global Cancer Statistics 2020: GLOBOCAN Estimates of Incidence and Mortality Worldwide for 36 Cancers in 185 Countries. CA Cancer J Clin.

[2] Xia F, Ndhlovu E, Zhang M, Chen X, Zhang B, Zhu P (2022). Ruptured hepatocellular carcinoma: current status of research. Front Oncol.

[3] Kudo M, Kitano M, Sakurai T, Nishida N (2015). General rules for the clinical and pathological study of primary liver cancer, nationwide follow-up survey and clinical practice guidelines: the outstanding achievements of the Liver Cancer Study Group of Japan. Dig Dis.

[4] Liberati A, Altman DG, Tetzlaff J, Mulrow C, Gøtzsche PC, Ioannidis JP, Clarke M, Devereaux PJ, Kleijnen J, Moher D (2009). The PRISMA statement for reporting systematic reviews and meta-analyses of studies that evaluate healthcare interventions: explanation and elaboration. BMJ.

[5] Buczkowski AK, Kim PT, Ho SG, Schaeffer DF, Lee SI, Owen DA, Weiss AH, Chung SW, Scudamore CH (2006). Multidisciplinary management of ruptured hepatocellular carcinoma. J Gastrointest Surg.

[6] Ou D, Yang H, Zeng Z, Luo Y, Yang L (2016). Comparison of the prognostic influence of emergency hepatectomy and staged hepatectomy in patients with ruptured hepatocellular carcinoma. Dig Liver Dis.

[7] Ren A, Luo S, Ji L, Yi X, Liang J, Wang J, Wan S (2019). Peritoneal metastasis after emergency hepatectomy and delayed hepatectomy for spontaneous rupture of hepatocellular carcinoma. Asian J Surg.

[8] Sun P, Song Z, Hu Q, Xiong J, Yang X, Zheng Q. Spontaneous rupture of hepatocellular carcinoma: a retrospective study of 87 patients in a teaching hospital. Chin-Ger J Clin Oncol. 2013;12:175–80. 10.1007/s10330-012-1112-8.

[9] Wu JJ, Zhu P, Zhang ZG, Zhang BX, Shu C, Mba'nbo-Koumpa AA, Zhang ZW, Huang ZY, Zhang WG, Lau WY, Chen XP (2019). Spontaneous rupture of hepatocellular carcinoma: optimal timing of partial hepatectomy. Eur J Surg Oncol.

[10] Yang H, Chen K, Wei Y, Liu F, Li H, Zhou Z, Li B (2014). Treatment of spontaneous ruptured hepatocellular carcinoma: A single-center study. Pak J Med Sci.

[11] Yang T, Sun YF, Zhang J, Lau WY, Lai EC, Lu JH, Shen F, Wu MC (2013). Partial hepatectomy for ruptured hepatocellular carcinoma. Br J Surg.

[12] Zhong F, Cheng XS, He K, Sun SB, Zhou J, Chen HM (2016). Treatment outcomes of spontaneous rupture of hepatocellular carcinoma with hemorrhagic shock: a multicenter study. SpringerPlus.

[13] Zhou C, Zhang C, Zu QQ, Wang B, Zhou CG, Shi HB, Liu S (2020). Emergency transarterial embolization followed by staged hepatectomy versus emergency hepatectomy for ruptured hepatocellular carcinoma: a single-center, propensity score matched analysis. Jpn J Radiol.

[14] Chearanai O, Plengvanit U, Asavanich C, Damrongsak D, Sindhvananda K, Boonyapisit S (1983). Spontaneous rupture of primary hepatoma: report of 63 cases with particular reference to the pathogenesis and rationale treatment by hepatic artery ligation. Cancer.

[15] Nagasue N, Inokuchi K (2010). Spontaneous and traumatic rupture of hepatoma. Br J Surg.

[16] Cherqui D, Panis Y, Rotman N, Fagniez PL (1993). Emergency liver resection for spontaneous rupture of hepatocellular carcinoma complicating cirrhosis. Br J Surg.

[17] Hai L, Yong-Hong P, Yong F, Ren-Feng L (2005). One-stage liver resection for spontaneous rupture of hepatocellular carcinoma. World J Surg.

[18] Liu CL, Fan ST, Lo CM, Tso WK, Poon RT, Lam CM, Wong J (2001). Management of spontaneous rupture of hepatocellular carcinoma: single-center experience. J Clin Oncol.

[19] Kwon JH, Song GW, Hwang S, Kim KH, Ahn CS, Moon DB, Ha TY, Jung DH, Park GC, Yoon YI, Shim JH, Kim KW, Lee SG (2021). Surgical outcomes of spontaneously ruptured hepatocellular carcinoma. J Gastrointest Surg.

[20] Lee HS, Choi GH, Kang DR, Han KH, Ahn SH, Kim DY, Park JY, Kim SU, Choi JS (2014). Impact of spontaneous hepatocellular carcinoma rupture on recurrence pattern and long-term surgical outcomes after partial hepatectomy. World J Surg.

