# Supplementary material for: Emergency versus delayed hepatectomy following transarterial embolization in spontaneously ruptured hepatocellular carcinoma survivors: a systematic review and meta-analysis
Source: World J Surg Oncol. 2022 Nov 18;20:365. doi: 10.1186/s12957-022-02832-7 (PMC9673318; doi:10.1186/s12957-022-02832-7)
Supplement: Supplementary file 1 — Additional file 1: Supplemental file 1. The search formula for each database. [file 12957_2022_2832_MOESM1_ESM.docx]

Supplemental file 1. The search formula for each database

The details are as follows:
Pubmed：("Hepatectomy"[MeSH Terms] OR "Hepatectomy"[All Fields] OR "hepatectomies"[All Fields] OR (("hepatic"[All Fields] OR "hepatophyta"[MeSH Terms] OR "hepatophyta"[All Fields] OR "hepatics"[All Fields]) AND ("resect"[All Fields] OR "resectability"[All Fields] OR "resectable"[All Fields] OR "resectates"[All Fields] OR "resected"[All Fields] OR "resecting"[All Fields] OR "resection"[All Fields] OR "resectional"[All Fields] OR "resectioned"[All Fields] OR "resectioning"[All Fields] OR "resections"[All Fields] OR "resective"[All Fields] OR "resects"[All Fields])) OR ("Hepatectomy"[MeSH Terms] OR "Hepatectomy"[All Fields] OR ("liver"[All Fields] AND "resection"[All Fields]) OR "liver resection"[All Fields]) OR "resection liver"[All Fields] OR "Hepatectomy"[MeSH Terms]) AND (("carcinoma, hepatocellular"[MeSH Terms] OR ("carcinoma"[All Fields] AND "hepatocellular"[All Fields]) OR "hepatocellular carcinoma"[All Fields] OR ("hepatocellular"[All Fields] AND "carcinoma"[All Fields]) OR "carcinoma, hepatocellular"[MeSH Terms]) AND ((("spontaneous"[All Fields] OR "spontaneously"[All Fields]) AND ("ruptur"[All Fields] OR "rupture"[MeSH Terms] OR "rupture"[All Fields] OR "ruptured"[All Fields] OR "ruptures"[All Fields] OR "rupturing"[All Fields])) OR ("rupture, spontaneous"[MeSH Terms] OR ("rupture"[All Fields] AND "spontaneous"[All Fields]) OR "spontaneous rupture"[All Fields] OR ("spontaneous"[All Fields] AND "rupture"[All Fields])) OR ("rupture*"[All Fields] AND "spontane*"[All Fields]) OR "rupture, spontaneous"[MeSH Terms]))

EMBASE: ('liver cell carcinoma'/exp OR 'carcinoma in the liver' OR 'carcinoma of the liver' OR 'carcinoma, hepatic cell' OR 'carcinoma, hepatocellular' OR 'carcinoma, liver' OR 'carcinoma, liver cell' OR 'hepatic carcinoma' OR 'hepatic cell carcinoma' OR 'hepato-carcinoma' OR 'hepato-cellular carcinoma' OR 'hepatocarcinoma' OR 'hepatocellular carcinoma' OR 'hepatocellular carcinomata' OR 'hepatocyte carcinoma' OR 'hepatocytic carcinoma' OR 'hepatoma' OR 'hepatomata' OR 'hepatomatous' OR 'liver carcinoma' OR 'liver carcinoma rupture' OR 'liver cell carcinoma' OR 'malignant hepatoma' OR 'primary liver carcinoma') AND 'spontaneously ruptured' AND ('hepatectomy'/exp OR 'hepatectomy' OR 'hepatic resection' OR 'liver resection' OR 'resection, liver') AND 'artificial embolization'/exp

Cochrane Library databases:

ID Search Hits

#1 MeSH descriptor: [Carcinoma, Hepatocellular] explode all trees 2028

#2 hepatocellular carcinoma 5690

#3 #1 OR #2 5690

#4 (Early OR emerge* OR one-stage):ti,ab,kw (Word variations have been searched) 185209

#5 (Delayed OR staged OR two-stage):ti,ab,kw (Word variations have been searched) 151075

#6 #4 OR #5 303562

#7 ('liver resection' OR hepatectomy):ti,ab,kw (Word variations have been searched) 4769

#8 embolization 2852

#9 #4 OR #5 303561

#10 MeSH descriptor: [Rupture, Spontaneous] explode all trees 113

#11 (ruptur*):ti,ab,kw (Word variations have been searched) 7558

#12 #10 OR #11 7558

#13 #3 AND #6 AND #9 AND #12 7
