# Supplementary material for: Emergency versus delayed hepatectomy following transarterial embolization in spontaneously ruptured hepatocellular carcinoma survivors: a systematic review and meta-analysis
Source: World J Surg Oncol. 2022 Nov 18;20:365. doi: 10.1186/s12957-022-02832-7 (PMC9673318; doi:10.1186/s12957-022-02832-7)
Supplement: Supplementary file 2 — Additional file 2: Supplemental file 2. The Risk of bias in the included retrospective cohort studies (by the Newcastle–Ottawa quality assessment tool). [file 12957_2022_2832_MOESM2_ESM.docx]

Supplemental file 2 the Risk of bias in the included retrospective cohort studies (by the Newcastle–Ottawa quality assessment tool)

| Study | Selection | | Comparability | | | | Outcome | | | Total |
| --- | --- | --- | --- | --- | --- | --- | --- | --- | --- | --- |
|  | 1 | 2 | 3 | 4 | 5 | 6 | 7 | 8 | 9 |  |
| Buczkowski 2006 | ☆ | ☆ | ☆ | ☆ | ☆ |  | ☆ | ☆ |  | 7 |
| Ou 2016 | ☆ | ☆ | ☆ | ☆ | ☆ |  | ☆ | ☆ | ☆ | 8 |
| Ren 2019 | ☆ | ☆ | ☆ | ☆ | ☆ |  | ☆ | ☆ |  | 7 |
| Sun 2013 | ☆ |  | ☆ | ☆ | ☆ |  | ☆ | ☆ |  | 6 |
| Wu 2019 | ☆ | ☆ | ☆ | ☆ | ☆ |  | ☆ |  |  | 6 |
| Yang H 2014 | ☆ | ☆ | ☆ | ☆ | ☆ |  | ☆ | ☆ |  | 7 |
| Yang T 2013 | ☆ | ☆ | ☆ | ☆ | ☆ |  | ☆ |  |  | 6 |
| Zhong 2016 | ☆ | ☆ | ☆ | ☆ | ☆ |  | ☆ |  |  | 6 |
| Zhou 2020 | ☆ | ☆ | ☆ | ☆ | ☆ | ☆ | ☆ | ☆ |  | 8 |

Note：1. Representativeness of exposed cohort; 2.Selection of non-exposed cohort; 3.Ascertainment of exposure; 4.Outcomeof interest was not present at start of study; 5. Study controls for age, sex, and marital status; 6. Study controls for any additional factors; 7.Assessment of outcomes; 8. Follow-up long enough for outcomes to occur; 9. Adequacy of follow-up.
