# Supplementary material for: Emergency versus delayed hepatectomy following transarterial embolization in spontaneously ruptured hepatocellular carcinoma survivors: a systematic review and meta-analysis
Source: World J Surg Oncol. 2022 Nov 18;20:365. doi: 10.1186/s12957-022-02832-7 (PMC9673318; doi:10.1186/s12957-022-02832-7)
Supplement: Supplementary file 3 — Additional file 3: Supplemental file 3. Begg’s test and Egger's test for each outcome. [file 12957_2022_2832_MOESM3_ESM.docx]

Supplemental file 3 Begg's test and Egger's test for each outcome

| Measured Outcomes | No. Studies | Begg's Test | | Egger's test |
| --- | --- | --- | --- | --- |
|  |  | Pr>\|z\|* | Pr > \|z\|** | P>\|t\| * |
| Hospital mortality | 8 | 0.188 | 0.26 | 0.107 |
| Postoperative complication | 6 | 0.327 | 0.462 | 0.077 |
| Postoperative hospital stay | 4 | 1 | 1 | 0.952 |
| Recurrence rate | 4 | 0.497 | 0.734 | 0.942 |
| Abdominal metastasis | 3 | 0.117 | 0.296 | 0.318 |
| 1-year OS | 6 | 0.573 | 0.707 | 0.429 |
| 3-year OS | 4 | 1 | 1 | 0.761 |

Note: *- P value; **- P value (continuity corrected); OS-overall survival; Statistical significant results are shown in bold.
